# The Relationship between Body Mass Index and Physical Fitness among Chinese University Students: Results of a Longitudinal Study

**DOI:** 10.3390/healthcare8040570

**Published:** 2020-12-17

**Authors:** Chen Ding, Yumei Jiang

**Affiliations:** School of Physical Education, Huazhong University of Science and Technology, Wuhan 430000, China; chending@hust.edu.cn

**Keywords:** body mass index (BMI), physical fitness, longitudinal data, overweight, obese

## Abstract

Over the past few decades, a gradual increase in sedentary lifestyles along with the increased consumption of a modern, hypercaloric diet has resulted in a substantial increase in the number of those classified as overweight or obese in China. The prevalence of overweight and obesity has become a key public health issue. However, it is important to be cautious when interpreting the literature as the majority of studies apply cross-sectional data to assess and subjectively compare the relationship between physical fitness and being overweight and obese. In the present study, longitudinal data were collected from 3066 students (enrolled in 2014) at a university in China at the beginning of each academic year throughout their four-year university program. The aim of this study was to analyze the various associations between BMI, explosive power, flexibility, and cardiorespiratory endurance, and a random-intercept panel model (RIPM) was separately employed on male and female participants to identify between- and within-person variations. In this way, the associations for between-person physical fitness and normal/overweight/obese weight ranges, and for within-person physical fitness and normal/overweight/obese weight ranges could be observed. The results of this study revealed that every physical fitness test chosen for evaluation (such as the standing long jump for explosive power or the distance run for cardiorespiratory endurance) was negatively related to the BMI results, irrespective of sex, with the notable exception of the flexibility results. In addition, this study showed that both males and females exhibited positively correlated results in both between-person BMI and flexibility as well as within-person BMI and flexibility. Furthermore, the relationships between and within persons of cardiorespiratory endurance, explosive power, and flexibility all showed positive correlations across both sexes. The dynamics between physical fitness and BMI identified in this study could prove useful to practitioners and researchers investigating such relationships in the future.

## 1. Introduction

Issues related to high numbers of overweight and obese people were once only of concern to the wealthy nations of the world; however, according to the World Health Organization (WHO), these concerns are now becoming more prevalent in poorer countries [1]. The WHO released a report in 2016 stating that 340 million 5 to 19-year-olds were overweight or obese; at the same time, the percentage of overweight adults over the age of 18 was reported to be 39%, while 13% were declared obese [1]. The rate of obesity has grown in all social and age groups over the last 20 years, and it has now become a familiar issue in both developed and developing countries [2,3].

Over the last few decades, China has undergone substantial economic reform, which has led to accelerated socioeconomic changes [4]. The increasing “modernization” of society and continuous improvements in the economy have been accompanied by an increasingly sedentary lifestyle and transformation of diet for many individuals [5,6]. Modern societal demands have also proven to have a detrimental effect on overall lifestyle due to added stress, which may further influence an individual’s BMI [7]. As a result, China has proven to be susceptible to the global trends of reduced levels of physical activity and increasing incidence of obesity across all age groups [8]. This increase in obesity has become a key public health issue as well as a growing concern that is increasingly being discussed by China’s Ministry of Education [9].

The WHO has confirmed that the body mass index (BMI) is a reliable and convenient way of assessing the relationship between an adult’s body mass and height [10]. Using BMI is an acceptable measurement for determining the health, physical fitness, and activity level of a given population that lacks regular high-intensity physical activity [11,12,13,14]. Therefore, the BMI metric will help to determine these factors in our sample, which consists of university students in China. It has been shown that high BMI scores often reflect a decrease in motor skills and a lower level of overall physical fitness in youth [15,16,17]. Fitness measures that require the projection of the body through space and movement such as running, jumping, and lifting or supporting the body off the ground are often impeded by the presence of excess weight or obesity [18,19]. In addition, the level of obesity is inversely correlated to the level of physical fitness in an individual [20,21]. As such, adults who are overweight or obese, when compared to the normal population, have a lower level of physical fitness [22]. More importantly, research concerning the relationships between BMI, physical activity, and physical fitness has demonstrated an empirically significant relationship between BMI and certain health parameters (e.g., [23,24]). For example, Adamu et al. [25] found that a lower level of physical fitness is not merely a risk factor that prevents physical activity itself, but it also exposes individuals to an increased risk of developing chronic diseases (e.g., cardiovascular diseases) as well as increased rates of mortality due to etiological factors. Thus, as the level of physical fitness declines, a rise in mortality is observed irrespective of BMI scores [26].

Research on the effects of weight status (normal and overweight/obese) on physical fitness has found a wide variety of results. For example, in a study by Bovet et al. [27] in which overweight and obese adolescents were compared with adolescents classified as normal weight, the adolescents of normal weight often performed at a higher level of physical fitness across a range of tests, excluding flexibility; Rauner et al. [28] found that overweight and obese participants showed decreased levels of physical fitness in tests for speed, cardiovascular function, and running endurance in comparison to participants in the normal weight range; and Artero et al. [29] reported that in a handgrip strength test, overweight adolescents outperformed their counterparts of normal weight. To account for this, the researchers suggested that muscle mass can develop to compensate for the added load caused by excessive fat stores [30]. Therefore, the results obtained for the overweight and obese participants who had higher levels of handgrip strength may be an instance of this phenomenon.

Although the literature on the relationship between BMI and levels of physical fitness is extensive, little attention has been paid specifically to Chinese college students. Furthermore, of these limited studies, descriptive studies are most predominant. For instance, Ni [31] found that the BMI values of the majority of university students in Shanghai ranged between the standard values of 18.5 to 24. However, the author points out that a considerable number of overweight students were identified, owing in part to unstructured, unhealthy diets, and sedentary lifestyles. Only a few studies have evaluated the correlation between BMI and physical fitness with regard to Chinese university students. In a study of 7234 students attending a university in Zhejiang province, China, Liu [32] found that students in the normal weight range performed better in the areas of cardiopulmonary function, speed, muscle strength, and endurance than their overweight and obese counterparts.

Previous research studying the connections between the weight categories “under-weight”, “normal weight”, “overweight”, and “obese” (according to BMI results) and physical fitness has provided valuable information for understanding the associated physical properties and individuals’ general health. However, cross-sectional data were used for these studies (e.g., [2,33,34]); more specifically, by collecting data over a determined period of time to assess and compare the relationship between results from normal-weight, overweight, and obese participants. These data very effectively represent between-person relationships between BMI scores and physical fitness; however, within-person relationships, which hold value in providing practitioners and researchers with crucial information with respect to developments in personal life [35], are not reflected in the data.

In order to understand the nature of within-person associations of participants classed as normal, overweight, or obese with their physical fitness, data should be collected at regular intervals over time; thus longitudinal studies are required. Such longitudinal studies can be difficult to come by.

One potential study, though, after testing a sample of Taiwanese students for explosive power, flexibility, and muscular endurance, Kung, Chang, Hwang, and Chi [36] explored the connections between the participants’ BMI results. The collected data were organized using the random-intercept panel model (RIPM) to display the variations in participant test results for both between- and within-person data sets. From this RIPM it could be deduced that irrespective of sex, those of a normal weight outperformed those of overweight and obese weight ranges in both explosive power and muscular endurance tests. From the results in the male model, female model, and combined model, an observable example of Simpson’s paradox could be recognized in the between-person relationships across BMI and explosive power (determined by a standing long- jump test), BMI and muscular endurance (determined by the number of sit-ups completed in 1 min), and BMI and flexibility (determined by a sit-and-reach test). This observation emphasized the importance of separately considering male and female results in future research. It is believed that studies not separately investigating male and female samples are at risk of arriving at incorrect or incomplete conclusions [36]. As data were only collected at the start and end of an academic year, the long-term differences between BMI and physical fitness were difficult to determine despite the effective methods of data collection utilized in the research by Kung et al.

In accordance with these recommendations from previous studies, the research in this study defined male and female samples to more accurately analyze the dynamic nature and processes between BMI and physical fitness. In particular, longitudinal data were collected from students at the beginning of each academic year throughout their four-year university programs in this study. This was done to identify variations between the between-person and within-person relationships with respect to weight ranges (e.g., normal and overweight/obese) and physical fitness. In this way, the connections found between between-person physical fitness and normal/overweight/obese weight ranges, and between within-person physical fitness and normal/overweight/obese weight ranges, can be observed without the aforementioned limitations identified in the previously discussed research.

To understand the ways normal/overweight/obese weight status in university student populations are related to between-person explosive power, flexibility, and cardiorespiratory endurance, as well as to within-person explosive power, flexibility, and cardiorespiratory endurance, the data analysis method used by Kung et al. [36] was applied in this study. It also utilized the random-intercept panel model (RIPM) as the means to analyze four sets of longitudinal data from a Chinese university. The between- and within-person variations were divided by the RIPM and organized into the variations of different cycles. By only taking results from one point in time, cross-sectional data are very difficult to model. This issue is resolved by between-person variations. In contrast, within-person variation exposed the relationships between within-person BMI and within-person flexibility, cardiorespiratory endurance, and explosive power [36]. The hypothesis is essentially that there are substantial connections across between- and within-level BMI and flexibility, BMI and cardiorespiratory endurance, BMI and explosive power, flexibility and cardiorespiratory endurance, flexibility and explosive power, and cardiorespiratory endurance and explosive power. Data collected from this approach can provide practitioners and researchers more informative results to better understand the nature of weight status and physical fitness; as a result, aspects of physical fitness and health of adolescents can be improved through the development of new and effective methods inspired by these findings, potentially improving individual’s management of their weight and preventing excessive weight gain [37].

## 2. Methods

### 2.1. Participants

This research sought to examine a population consisting of Chinese university students. The authors used data collected from the standards of the national student fitness test (NSFT) of the Ministry of Education of the People’s Republic of China conducted over a period of four years (i.e., 2014 to 2018) at this university. The first class of students to be tested included all students enrolling in undergraduate programs in 2014. Field-based physical fitness tests and objective height and weight measurements were performed and collected from participants. The same sample of students repeated these tests in the Fall of 2015 (sophomore), 2016 (Junior), and 2017 (Senior). To ensure the reliability, authenticity, and effectiveness of the data researchers excluded data provided by students who did not finish all four years of tests. Researchers screened out the physical fitness assessment data of 3066 students (2186 males and 880 females) for study and analysis. The disproportionate ratio between male and female participants in the sample is consistent with the discrepancies observed in the gender of students enrolling in scientific and technological university programs at the university.

### 2.2. Measurements

#### 2.2.1. Body Mass Index

Body mass index (BMI) is a universally used unit of measurement expressed as kg/m^2^. It is therefore calculated by dividing an individual’s mass in kilograms by the square of their height in meters. The World Health Organization (WHO) has provided the following values most commonly used to help determine an individual’s weight status according to BMI: underweight individuals score below 18.5 kg/m^2^; those classed as having normal weight score between 18.5 and 23.9 kg/m^2^; overweight individuals receive scores between 24 and 27.9 kg/m^2^; individuals classed as obese could score anything over 28 kg/m^2^ [8]. Students’ weight and height were measured annually so that the BMI of participants could be calculated. Students who could be classified as being of normal weight, overweight, or obese were selected as participants for this study.

#### 2.2.2. Explosive Power: Standing Long Jump

From a standing position at the starting line, each participant was directed to jump forward off both feet as far horizontally as possible. Participants were permitted to crouch before launching themselves. The distance recorded was from the take-off mark and measured to the back of the closest foot. After two attempts, the greatest distance was retained for analysis.

#### 2.2.3. Flexibility: Sit and Reach

Participants extended their hands towards their feet in a seated position with fingertips touching a ruler set at the feet. The furthest point reached was rounded to the nearest centimeter and recorded. Shoes were taken off before the test, and participants were directed to fully extend their knees and slowly reach forward along the 25 cm scale set from the bottom of the participant’s feet. The participants could make two attempts, and the better result was collected for data analysis.

#### 2.2.4. Cardiorespiratory Endurance: 800/1000 m Run/Walk

The test was measured in minutes and seconds. Participants were provided with time to adequately warm up before the test. Subjects in a group of six to eight were directed to stand behind a starting line upon hearing the investigator call, “Take your marks”. At the word, “go”, the participants were to begin the 800 m or 1000 m walk or run. The participants were instructed to maintain a steady pace while trying to finish the test as quickly as possible. If required, the participant could choose to walk if a running pace could not be maintained. All female students ran or walked an 800 m course, and male students walked or ran a 1000 m course.

### 2.3. Statistics and Analysis

The structural equation model was utilized when examining the longitudinal links between participants’ BMI results and their performance in explosive power, flexibility, and cardiorespiratory endurance tests. To represent the relationships between BMI, explosive power, flexibility, and cardiorespiratory endurance, the researchers developed the RIPM. BMI, explosive power, flexibility, and cardiorespiratory endurance results were each individually modeled into between- and within-person variations. The influences on between-person BMI and each between-person physical fitness test result could thereby be examined. In addition, this method allowed for the examination of the differences between within-person BMI, flexibility, cardiorespiratory endurance, and explosive power. These models were generated using the Mplus 8.2 software (Muthén & Muthén, Los Angeles, CA, USA).

Various goodness-of-fit statistics and the Chi-Square value were used to determine the overall model fit. In particular, the goodness-of-fit statistics include the comparative fit index (CFI) ≥ 0.95 [38], root mean square error of approximation (RMSEA) ≤ 0.08 [34], non-normed fit index (NNFI) > 0.95 [39], and standardized root mean square residual (SRMR) ≤ 0.08 [40]. Values of correlation coefficients between BMI, explosive power, flexibility, and cardiorespiratory endurance less than 0.24 were considered as representing low correlation, while values of correlation coefficients exceeding 0.24 indicated a moderate correlation [36].

## 3. Results

As shown in Table 1, the distribution of BMI values of participants was basically normal. However, it should be noted that there was a certain proportion of subjects who were either overweight or obese. Male students in each grade whose BMI fell into the normal range accounted for 70.7% to 77.6% of the total subjects, and their BMI values showed an inverted U-shaped trend. The proportion of BMI values in the normal range increased in the first two academic years but dropped in the latter. Similarly, female students whose BMI values fell within the normal range accounted for the vast majority of the subjects, between 80.3% to 86.6%. The overweight and obesity rate for male students was higher than for females. One particular difference observed across the male students was that the proportion of BMI values in the normal range increased in the first three academic years only to drop in the last.

In Table 2, the mean, standard deviation, and *t* value of the main variables for the male and female participants are defined. The *t* values of the male participants show that BMI and explosive power were greater than those of their female counterparts. The female participants on the other hand performed better overall in the flexibility tests each year.

Table 3 shows BMI, explosive power, flexibility, and cardiorespiratory endurance coefficients for participants with normal/overweight/obese weight status. For both male and female participants, the BMI results recorded each year were closely correlated with themselves (0.71 to 0.94; 0.64 to 0.87), which was not unlike the coefficients for explosive power (0.60 to 0.73; 0.60 to 0.77), flexibility (0.50 to 0.69; 0.58 to 0.76), and cardiorespiratory endurance (0.46 to 0.65; 0.40 to 0.63).

For male participants across the four-year testing period, a small negative correlation existed between BMI and explosive power (−0.14 to −0.21) (Table 3). The BMI and flexibility of the male participants over the four years exhibited only a weak positive correlation (0.01 to 0.09), while BMI and cardiorespiratory endurance also showed a weak to moderate positive correlation (0.11 to 0.31) across the four rounds of testing. The results of the running test are based on the time required by participants to complete the test and were utilized to assess cardiorespiratory endurance. As such, a positive correlation between cardiorespiratory endurance and BMI reflects the innately negative nature of the two variables.

Male explosive power and flexibility (0.03 to 0.12) over the four years of tests showed a positive and small correlation, whereas explosive power and cardiorespiratory endurance (−0.14 to −0.33) had a small to moderate negative correlation, and flexibility and cardiorespiratory endurance exhibited a small and negative correlation (0.04 to −0.07) (Table 3).

After analyzing the relationship between the female participants’ results, a small and negative correlation between BMI and explosive power (0.02 to −0.13) was revealed over the testing period. In contrast, BMI and flexibility (−0.01 to 0.06) showed a small zero to positive correlation in the four-years of testing. For BMI and cardiorespiratory endurance (0.03 to 0.18), a small and positive correlation was found, as was also found between explosive power and flexibility (0.02 to 0.12) in the four tests. A small to moderate negative correlation was observed for female explosive power and cardiorespiratory endurance results (−0.13 to −0.29), while for flexibility and cardiorespiratory endurance (−0.01 to −0.08), a small and negative correlation was found. 

**Figure 1:**
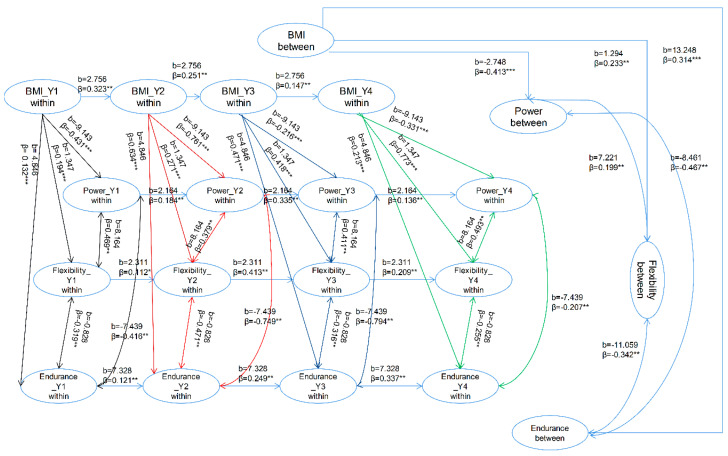
Estimated random intercept panel model of the male samples: simplified and represented above. * *p* < 0.05; ** *p* < 0.01, *** *p* < 0.001.

The model representing the male results shows an appropriate goodness of fit (χ^2^ (17) = 33.79, *p* < 0.001, RMSEA = 0.058, CFI = 0.99, SRMR = 0.023). Figure 1 shows estimates of the coefficients of the male model and the between-person relationship between BMI and explosive power. These coefficients were b = −2.748, β = −0.413, *p* < 0.001. According to these results, for male university students of normal, overweight, or obese body compositions, a higher BMI indicates a lower level of explosive power. For the between-person relationship between BMI and flexibility, the coefficients b = 1.294, β = 0.233, *p* < 0.01 were found, suggesting that for higher results in between-person BMI, flexibility results were also generally higher. The between-person BMI and cardiorespiratory endurance relationship revealed the coefficients to be b = 13.248, β = 0.314, *p* < 0.001, implying that participants with a higher BMI result required more time to finish the running test and therefore exhibited poorer performance in their overall level of cardiorespiratory endurance. The between-person relationship between explosive power and flexibility revealed a significantly positive correlation, as seen with the modeled coefficients of b = 7.221, β = 0.199, *p* < 0.01. For the between-person association of explosive power and cardiorespiratory endurance, the coefficient of the model was b = −8.461, β = −0.467, *p* < 0.01. These results demonstrate that the participants with greater explosive power regularly performed the running test much more quickly, thus using less time, demonstrating a greater level of cardiorespiratory endurance. This explains the significantly negative correlation between these results: as explosive power increases, so does cardiorespiratory endurance. For the between-person association between flexibility and cardiorespiratory endurance, the coefficient of the model was b = −11.059, β = −0.342, *p* < 0.01 implying that the participants who had recorded a higher level of flexibility often returned better levels of cardiorespiratory endurance, requiring less time to finish the running test.

Within-person BMI and explosive power results for male participants were represented in the model with the coefficients b = −9.143, βs = −0.216 to −0.761, *p* < 0.001 (Figure 1). These results reveal that, for male participants, as the within-person BMI increases, within-person explosive power decreases.

The association between the within-person BMI and flexibility in male participants was defined by the coefficients b = 1.347, βs = 0.194 to 0.773, *p* < 0.001 (Figure 1), suggesting that male participants with greater within-person BMI results corresponded to improved flexibility results

For within-person BMI and cardiorespiratory endurance, the coefficients of male models were b = 4.848, βs = 0.132 to 0.471, *p* < 0.001 (Figure 1), showing that for male participants, those with a higher BMI needed more time to finish the running test; in other words, they had poorer cardiorespiratory endurance.

The coefficients representing the male participants’ within-person association between explosive power and flexibility were b = 8.164, βs = 0.379 to 0.493, *p* < 0.05 (Figure 1), showing that for the male university students, higher within-person explosive power corresponded to a higher level of within-person flexibility. For the association between within-person explosive power and cardiorespiratory endurance, the coefficients of male models were b = −7.439, βs = −0.207 to −0.794, *p* < 0.05 (Figure 1), indicating that those with higher levels of explosive power often performed better in the running test and had a higher level of cardiorespiratory endurance.

The relationship between within-person flexibility and within-person cardiorespiratory endurance for male participants was able to be modeled with the coefficients of b = −0.828, βs = −0.255 to 0.471, *p* < 0.05 (Figure 1), showing that, for male participants, greater within-person explosive power suggested a higher level of within-person cardiorespiratory endurance.

**Figure 2:**
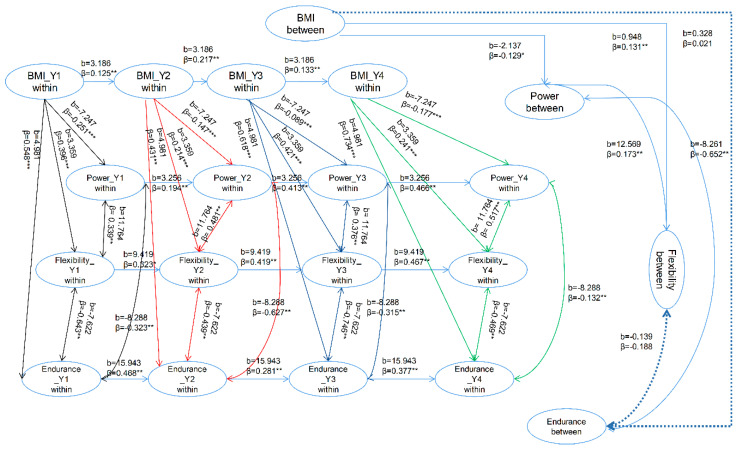
Estimated random intercept panel model of the female samples: simplified and represented above. The dotted line indicates the statistically insignificant coefficient. * *p* < 0.05; ** *p* < 0.01, *** *p* < 0.001.

The model representing the female results shows an appropriate goodness of fit (χ^2^ (17) = 30.61, *p* < 0.001, RMSEA = 0.046, CFI = 0.99, SRMR = 0.015). Figure 2 provides the estimated coefficients of the female model. The association between between-person BMI and between-person explosive power in the female model was described by the coefficients b = −2.137, β = −0.129, *p* < 0.05. As with the male participants, the female university students who held normal, overweight or obese body composition classifications showed that as BMI increased, explosive power test results decreased. Similar relationships were found for male and female participants for the between-person association across BMI and flexibility, with the female model being defined by the coefficients b = 0.948, β = 0.131, *p* < 0.01. As the between-person BMI results for female participants increased, their between-person flexibility results also increased. The between-person BMI and cardiorespiratory endurance relationship was represented with the modeled coefficient of b = 0.328, β = 0.021, *p* > 0.05. Although this result is not statistically significant, it mildly suggests that as the female participants’ between-person BMI increased, more time was required to finish the running test, and while this is a positive correlation, more time represents a lower level of cardiorespiratory endurance. The between-person relationship between explosive power and flexibility had a coefficient of the model of b = 12.569, β = 0.173, *p* < 0.01. This reveals a strong positive correlation between flexibility and explosive power results. For the relationship between the between-person explosive power and cardiorespiratory endurance, the coefficients of the model were b = −8.261, β = −0.652, *p* < 0.01. These results, not unlike the male results, represent a significantly negative correlation in the relationship between explosive power and cardiorespiratory endurance, thereby indicating that superior explosive power often results in a shorter time, representing a better cardiorespiratory endurance result. Unlike the male between-person association between flexibility and cardiorespiratory endurance, the female coefficient of the model was b = −0.139, β = −0.188, *p* = 0.06. These results, while significant for the male participants, demonstrated a rather insignificant negative correlation for the female university students.

The relationship between within-person BMI and within-person explosive power was represented by the coefficients b = −7.247, βs = −0.089 to −0.251, *p* < 0.001 (Figure 2). These results revealed that, similarly to the male university students, for female participants, as their within-person BMI increased, within-person explosive power decreased.

Within-person BMI and flexibility for females showed coefficients of b = 3.359, βs = 0.214 to 0.418, *p* < 0.001 (Figure 2), suggesting that in the female model, as the within-person BMI increased, so too did the within-person flexibility.

The relationship between female within-person BMI and cardiorespiratory endurance was modeled with the coefficients b = 4.981, βs = 0.431 to 0.734, *p* < 0.001 (Figure 2), showing that female participants with higher within-person BMI results used more time to complete the running tests, demonstrating a poorer level of cardiorespiratory endurance.

Female university students who scored higher within-person explosive power results often also performed better in the sit-and-reach test. This association is represented by the coefficients representing the relationship between explosive power and flexibility as b = 11.764, βs = 0.339 to 0.517, *p* < 0.05 (Figure 2). The within-person association between explosive power and cardiorespiratory endurance shared similar results with the male participant coefficients with b = −8.288, βs = −0.132 to −0.627, *p* = 0.03 (Figure 2), indicating that those who scored higher within-person explosive power results performed better on the running test and thus had higher levels of cardiorespiratory endurance.

The coefficients modeled to represent within-person flexibility and cardiorespiratory endurance for female participants were b = −7.622, βs = −0.439 to 0.746, *p* = 0.02 (Figure 2), showing that female participants with greater explosive power consistently demonstrated a higher level of cardiorespiratory endurance.

## 4. Discussion

The results of this study show that participants demonstrated a relatively centralized distribution of BMI values that were generally within the normal range (BMI = 18.5–23.9 kg/m^2^). In terms of students with body shapes influenced by an unhealthy diet or lifestyle, there were some overweight and even obese students. The overweight and obesity rate for male students was higher than for females. The difference in the distribution of BMI between male and female students may be due to female students placing a greater concern on their weight and body image compared to their male counterparts [41].

In addition, the results of this study revealed that overweight and obese students all had poorer performance in both the standing long jump and distance running physical fitness tests compared with their normal-weight counterparts regardless of sex, which is consistent with previous research results [37,42,43,44]. Specifically, this study’s findings showed that BMI levels negatively correlated with the scores taken from the standing long jump test, which was used to measure explosive power, regardless of the sex of the participant. This relationship was also observed in other research, which found that overweight or obese participating adolescents tested more poorly in the explosive power test compared to those within a normal weight range (e.g., [37,45]). As for the relationship between participant cardiorespiratory endurance and weight status (underweight, normal, overweight/obese), in both male and female participants, it was found that as between-person BMI increased, cardiorespiratory endurance decreased, demonstrating that between-person BMI and cardiorespiratory endurance have a negative correlation. This study discovered, however, that statistically, the results used to define the male participant model showed a strong correlation. On the other hand, the female model revealed a statistically insignificant correlation between between-person BMI and cardiorespiratory endurance. Likewise, Chen, Cui, Zhang, and Peng [8] reported lower levels of distance running performance among participants of overweight or obese status than their counterparts of a normal weight range. Some possible explanations by other researchers for the poorer performance of physical fitness observed in overweight and obese students include the fact that the additional load and restriction of movement caused by excess body mass itself can impede performance; the greater need for energy to perform physical activities with such loads (compared to those of a normal range) can cause students to avoid physical activity. On an emotional level, poor performance can diminish the confidence of an individual, potentially leading to anxiety or depression; the resulting negative feelings may also reduce the individual’s motivation or willingness to participate in physical activity [46,47,48].

The relationship between BMI and flexibility, which was assessed with the sit-and-reach test, revealed conflicting results between different studies. The results from Chen et al. [43], Mak et al. [44] and Prista, Maia, Damasceno, and Beunen [49] can be taken as examples: Chen et al. revealed that BMI and flexibility test results showed a negative relationship; Mak et al. [44] reported that participants classified as normal, overweight, and obese all returned similar results in the sit-and-reach test; Prista et al. reported that participants of a normal weight range performed slightly worse than those of an overweight or obese body composition. The present study’s findings share some consistency with the reported results found by Kung et al. [36]. This study shows that both males and females exhibited positive correlations in both between-person BMI and flexibility as well as between within-person BMI and flexibility. In addition, Kung et al. [36] revealed that the between-person BMI and flexibility results of the male participants correlated positively, whereas the female participant results correlated negatively. Further still, the correlation between within-person BMI and flexibility results was often negative, irrespective of the participants’ sex. It can be concluded, then, that body mass has little impact on an individual’s flexibility.

In accordance with previous research, the between- and within-person explosive power, flexibility, and cardiorespiratory endurance relationships revealed in this study, irrespective of participant sex, all showed positive correlations. The conclusions coincide with those determined by He et al. [45], who derived significant correlations between handgrip strength tests, vertical jump tests, and sit-and-reach tests for both male and female participant performance results. When compared with their normal-weight counterparts, the poorer performance observed in students of overweight and obese ranges could be accounted for in a ratio comparing muscle mass with total body mass; after all, fitness tests often take both physical strength and body mass into consideration [27].

## 5. Limitations and Suggestions for Future Studies

Although the research conducted in this study was relatively successful, there are always limitations to research. To begin with, the results completely excluded participants who may be identified as underweight, only selecting for university students of average weights, students who are overweight, and those who are obese. Therefore, the relationship between underweight participants and their ability to perform in flexibility, explosive power, and cardiorespiratory endurance tests remains unexplored. In addition, this study does not discuss the correlation of BMI with other aspects of physical fitness beyond flexibility, explosive power, and cardiorespiratory endurance of participants. Using additional tests to analyze the association between BMI and physical fitness could provide even deeper findings from which different conclusions may be derived. In future research, it will be important to include more items to test different aspects of physical fitness.

## 6. Conclusions and Implication

Based on the above results, it can be concluded that there were obvious stages of change in the BMI results of these university students during their time in school; these were probably influenced by the physical education curriculum. In the first two years, when physical education was compulsory, regular exercise helped these university students improve and maintain their physical fitness; the absence of physical education in the curriculum in the last two years and a general lack of awareness of the importance of exercise explains their significant decline in physical fitness in their latter two years in school. On the one hand, these results demonstrate the significant achievement of the physical education curriculum. On the other hand, the problems are clear: juniors and seniors are devoting inadequate attention to physical exercise; there is a lack of conscious awareness of the importance of physical activity and an absence of guidance from the educational institution with regards to students’ physical wellbeing. The purpose of including sports in schools is to encourage a certain level of physical fitness in college students, and thus, it makes up a key component of physical education in schools. By focusing on physical fitness, active and habitual participation in exercise can be promoted to students, allowing them to better understand their own overall level of physical health and fitness [8].

In addition, the dynamics between physical fitness and BMI identified in this study could prove useful to practitioners and researchers investigating such relationships in the future. As per the findings of this study, the physical ability of university students is impaired as BMI increases beyond an average weight range. This negative influence not only affects explosive power and flexibility but also impacts cardiorespiratory endurance. This study shows that low levels of fitness (particularly seen in overweight or obese individuals) are associated with adverse health effects. It is important to remedy this issue most obviously by increasing physical activity. However, as mentioned, those with excess body mass may find their body mass itself an obstacle, making physical activity more difficult to engage in when compared to those of a normal weight range. A potential cycle may be observed in students who fear that their poor performance may lead to social rejection, causing them to be more reluctant to participate in physical activity, in turn leading to a failure to develop physical skills and fitness to succeed in the future. Such complex anxieties may require expert social, psychological, and technical support to break the cycle and increase the accessibility of physical activity, encouraging and supporting participation for those who need it most [34]. It is recommended to increase university students’ awareness of the importance of regular physical activity, helping students make informed health choices and develop better exercise habits to maintain their overall physical ability and a healthy physique. As a way to support their students, universities should offer a variety of physically active extracurricular activities to help encourage students to develop the aforementioned physical activity habits [50]. Through the formation of more sports clubs and use of the College Students Sports Association, and with a wider variety for sporting completions and activities, students can find pleasure in cultivating healthy routines and increasing exposure to environments conducive to developing a greater awareness of the positive impacts physical activity has on day-to-day life [51].

## Figures and Tables

**Table 1:** BMI distribution according to sex over four years.

	Normal		Overweight		Obese	
	Male	Female	Male	Female	Male	Female
BMI_Y1 category, *n* (%)	1578 (72.2)	731 (83.1)	468 (21.4)	133 (15.1)	140 (6.4)	16 (1.8)
BMI_Y2 category, *n* (%)	1697 (77.6)	707 (80.3)	393 (18.0)	156 (17.7)	96 (4.4)	17 (1.9)
BMI_Y3 category, *n* (%)	1598 (73.1)	762 (86.6)	461 (21.1)	104 (11.8)	127 (5.8)	14 (1.6)
BMI_Y4 category, *n* (%)	1546 (70.7)	744 (84.5)	497 (22.7)	115 (13.1)	143 (6.5)	21 (2.4)

**Table 2:** Means, standard deviations (SD), and sample size (N) of main variables.

	Male (*n* = 2186)		Female (*n* = 880)		*t*	Value (*p*)
	Mean	SD	Mean	SD		
BMI_Y1 kg/m^2^, mean (sd)	22.65	3.54	21.68	2.40	7.47	(*p* < 0.001)
BMI_Y2 kg/m^2^, mean (sd)	22.53	2.60	22.22	2.25	3.14	(*p* = 0.002)
BMI_Y3 kg/m^2^, mean (sd)	22.78	2.82	21.73	2.73	9.39	(*p* < 0.001)
BMI_Y4 kg/m^2^, mean (sd)	22.98	2.88	21.78	2.34	10.91	(*p* < 0.001)
Power_Y1 cm, mean (sd)	219.63	17.20	162.71	16.03	84.50	(*p* < 0.001)
Power_Y2 cm, mean (sd)	220.59	17.96	163.89	14.94	82.83	(*p* < 0.001)
Power_Y3 cm, mean (sd)	220.32	17.80	163.60	15.04	83.32	(*p* < 0.001)
Power_Y4 cm, mean (sd)	223.18	17.65	167.16	15.30	82.51	(*p* < 0.001)
Flexibility_Y1 cm, mean (sd)	12.70	6.56	14.81	5.98	−8.27	(*p* < 0.001)
Flexibility_Y2 cm, mean (sd)	13.73	6.07	15.50	5.48	−7.52	(*p* < 0.001)
Flexibility_Y3 cm, mean (sd)	14.23	5.82	17.68	5.61	−14.98	(*p* < 0.001)
Flexibility_Y4 cm, mean (sd)	14.54	5.81	17.75	5.64	−13.98	(*p* < 0.001)
Endurance_Y1 km/h, mean (sd)	16.09	1.60	13.26	1.19	47.60	(*p* < 0.001)
Endurance_Y2 km/h, mean (sd)	16.22	1.56	13.29	1.17	50.43	(*p* < 0.001)
Endurance_Y3 km/h, mean (sd)	14.97	1.73	12.17	1.35	42.86	(*p* < 0.001)
Endurance_Y4 km/h, mean (sd)	14.67	1.77	11.86	1.36	42.18	(*p* < 0.001)

Note: Y1 = first year test; Y2 = second year test; Y3 = third year test; Y4 = fourth year test.

**Table 3:** Coefficients of BMI, flexibility, explosive power, and cardiorespiratory endurance with normal/overweight/obese weight status.

	BMI _Y1	BMI _Y2	BMI _Y3	BMI _Y4	Power _Y1	Power _Y2	Power _Y3	Power _Y4	Flexibility_Y1	Flexibility_Y2	Flexibility_Y3	Flexibility_Y4	Endurance_Y1	Endurance_Y2	Endurance_Y3	Endurance_Y4
BMI_Y1	1.00	0.87 **	0.64 **	0.79 **	−0.06	−0.10 **	−0.09 **	−0.10 **	0.02	0.01	0.03	0.02	0.13 **	0.14 **	0.03	0.04
BMI_Y2	0.76 **	1.00	0.70 **	0.84 **	−0.06	−0.12 **	−0.09 **	−0.11 **	0.01	0.02	0.06	0.05	0.11 **	0.15 **	0.03	0.05
BMI_Y3	0.72 **	0.87 **	1.00	0.74 **	0.02	−0.03	−0.04	−0.05	0.02	0.02	0.01	0.01	0.09 *	0.13 **	0.06	0.04
BMI_Y4	0.71 **	0.85 **	0.94 **	1.00	−0.07	−0.12 **	−0.11 **	−0.13 **	0.02	−0.01	−0.02	−0.03	0.13 **	0.18 **	0.11 **	0.08 *
Power_Y1	−0.17 **	−0.15 **	−0.17 **	−0.17 **	1.00	0.62 **	0.62 **	0.60 **	0.05	0.09 **	0.02	0.04	−0.29 **	−0.22 **	−0.18 **	−0.13 **
Power_Y2	−0.21 **	−0.20 **	−0.20 **	−0.20 **	0.68 **	1.00	0.71 **	0.71 **	0.07 *	0.10 **	0.05	0.06	−0.30 **	−0.25 **	−0.21 **	−0.14 **
Power_Y3	−0.16 **	−0.15 **	−0.20 **	−0.19 **	0.61 **	0.67 **	1.00	0.77 **	0.07 *	0.12 **	0.08 *	0.06	−0.31 **	−0.24 **	−0.25 **	−0.17 **
Power_Y4	−0.15 **	−0.14 **	−0.20 **	−0.22 **	0.60 **	0.67 **	0.73 **	1.00	0.04	0.09 **	0.07 *	0.10 **	−0.27 **	−0.23 **	−0.21 **	−0.15 **
Flexibility_Y1	0.05 *	0.09 **	0.08 **	0.08 **	0.05 *	0.03	0.06 *	0.07 **	1.00	0.58 **	0.58 **	0.58 **	−0.05	−0.02	−0.04	−0.04
Flexibility_Y2	0.01	0.01	0.01	0.02	0.10 **	0.13 **	0.10 **	0.11 **	0.55 **	1.00	0.68 **	0.64 **	−0.08 *	−0.01	−0.04	−0.01
Flexibility_Y3	0.03	0.05 *	0.02	0.03	0.08 **	0.10 **	0.12 **	0.12 **	0.52 **	0.59 **	1.00	0.76 **	−0.05	−0.03	−0.08 *	−0.06
Flexibility_Y4	0.04	0.06 **	0.05 *	0.04	0.05 *	0.11 **	0.09 **	0.12 **	0.50 **	0.58 **	0.69 **	1.00	−0.03	0.00	−0.06	−0.04
Endurance_Y1	0.31 **	0.30 **	0.31 **	0.29 **	−0.29 **	−0.33 **	−0.29 **	−0.27 **	0.04	−0.04	−0.04	−0.01	1.00	0.63 **	0.51 **	0.40 **
Endurance_Y2	0.28 **	0.28 **	0.29 **	0.27 **	−0.25 **	−0.29 **	−0.27 **	−0.25 **	−0.02	−0.06 **	−0.07 **	−0.04	0.65 **	1.00	0.55 **	0.45 **
Endurance_Y3	0.15 **	0.17 **	0.20 **	0.19 **	−0.21 **	−0.23 **	−0.23 **	−0.24 **	0.00	−0.04	−0.07 **	−0.01	0.52 **	0.57 **	1.00	0.60 **
Endurance_Y4	0.11 **	0.13 **	0.16 **	0.15 **	−0.14 **	−0.14 **	−0.17 **	−0.15 **	−0.02	−0.05 *	−0.10 **	−0.03	0.46 **	0.50 **	0.65 **	1.00

Note: The upper and lower triangular sections show the female and male samples, respectively; * *p* < 0.05; ** *p* < 0.01.
